# Chromosome gains drive childhood ALL

**DOI:** 10.18632/oncotarget.5141

**Published:** 2015-08-10

**Authors:** Kajsa Paulsson

**Affiliations:** Department of Laboratory Medicine, Division of Clinical Genetics, Lund University, Lund, Sweden

**Keywords:** Chromosome Section, aneuploidy, hyperdiploidy, leukemia, chromosomal instability

High hyperdiploid (51-67 chromosomes) acute lymphoblastic leukemia (ALL), characterized genetically by a nonrandom gain of chromosomes, is one of the most common childhood malignancies. It is associated with a B-cell precursor immunophenotype and shows a distinct age peak at 2-4 years, with adult cases being much less common. The gained chromosomes generally forms a specific pattern of trisomies X, 4, 6, 10, 14, 17, and 18, and tri- or tetrasomy 21, with no concurrent monosomies [1]. In contrast to the majority of aneuploid solid tumors, which generally displays an increased rate of gains and losses of whole chromosomes termed chromosomal instability (CIN), there is no evidence of ongoing CIN in high hyperdiploid childhood ALL. Instead, these cases generally have no or few subclones, no clonal evolution between diagnosis and relapse samples as regards chromosomal content, and little cell-to-cell heterogeneity on the chromosomal level [1].

We recently published a whole genome and exome sequencing study of 51 cases of high hyperdiploid childhood ALL, characterizing the genomic landscape of this disease [2]. This investigation showed that high hyperdiploid cases harbored relatively few other genetic mutations, with no recurrent fusion gene and only 7.5 point mutations on average in coding regions. Putative driver events besides the aneuploidy comprised mutations in the RTK/RAS pathway, such as *KRAS*, and in histone modifiers, in particular *CREBBP*. Notably, such mutations have been shown to frequently be subclonal and to arise or disappear at relapse, whereas the chromosomal gains usually are stable [3;4]. Taken together with the fact that RTK/RAS and histone modifier mutations were only seen in a subset of high hyperdiploid cases, we concluded that the gained chromosomes are the primary driver event in this form of pediatric ALL [2].

How and when, then, do the chromosomal gains occur? As to the “how”, this process is still an enigma, considering the apparent lack of CIN in high hyperdiploid childhood ALL. Notably, both parental homologues are always duplicated in chromosomes that have gained two copies, suggesting that the extra copies were gained in the same cell division and not sequentially. Together with data on the frequency of uniparental disomies, this indicates that the high hyperdiploid pattern likely arose in a single abnormal mitosis of a diploid cell [1]. To see whether we could determine when this mitosis occurred, we looked at the mutant allele fractions of mutations in trisomic chromosomes, including both putative driver and passenger mutations. We assumed that a mutation that was present in two of the three chromosomal copies most likely arose before the duplication of one homologue, whereas mutations that were present in one of three chromosomal copies could have arisen either before (if the other homologue was subsequently duplicated) or after the duplication. In this way, we could study the temporal sequence of mutational events in high hyperdiploid childhood ALL. The analysis clearly showed that the trisomies, and hence most likely the high hyperdiploid pattern in its entirety, arose very early in most cases [2]. This agrees well with previous indications that high hyperdiploid cells may arise very early in life in children who years later develop ALL, based on backtracking studies of *IGH* and *TCR* rearrangements from the leukemic clone to neonatal blood spots, twins with concurrent high hyperdiploid childhood ALL of a shared origin, and one case study where trisomic cells could be found in stored cord blood [5–7]. Thus, the “typical” high hyperdiploid ALL case may have acquired chromosomal gains prenatally, gone through a stage of preleukemia where the leukemic cells presumably obtained other genetic aberrations - most likely mutations in the RTK/RAS pathway and/or in histone modifiers - and eventually present as full-blown leukemia when the patient reaches 2-4 years of age. Interestingly, the only two cases in our investigation that appeared to have gained the extra chromosomes relatively late in the temporal order of events were the two oldest patients investigated [2]; both were 13 years old at diagnosis and thus untypical as regards their ages, since the vast majority of cases of high hyperdiploid childhood ALL occurs in children below ten years of age. One intriguing possibility is therefore that the chromosomal gains may have different origins in young and older patients, something that may partly explain the marked age peak in early childhood of these cases. In the future, similar investigations of adolescent and adult cases, as well as paired diagnosis and relapse samples from pediatric cases, may yield further insight into the origin of the chromosomal gains of high hyperdiploid ALL and increase our understanding of aneuploid malignancies not associated with CIN.
